# Physicians’ Perspectives, Well-being, and Fulfilment in Telemedicine: A Cross-Sectional Study

**DOI:** 10.1089/tmr.2024.0005

**Published:** 2024-04-25

**Authors:** Cristina Sicorschi Gutu, Krisztina Schmitz-Grosz, Carsten Sommer-Meyer, Günter Niklewski

**Affiliations:** ^1^ Medgate AG, Basel, Switzerland.; ^2^ University Clinic for Psychiatry and Psychotherapy, Paracelsus Medical University, Nuremberg, Germany.

**Keywords:** telemedicine, primary care, mental health, work–life balance

## Abstract

**Background:**

Telemedicine, a safe way of medical care appreciated by patients, has proven to have different benefits for our health care systems. Studies on patient experiences and outcomes in telemedicine are thriving. However, few studies have explored the possible associations between telemedicine and physician work–life balance or mental health.

**Methods:**

A cross-sectional survey for physicians who practiced in Switzerland was conducted. Physicians who participated answered validated questions about work–life balance, professional satisfaction, emotional exhaustion, and depression. There were three groups a control group with physicians who did not practice telemedicine (*n* = 30), an experimental group with physicians who practiced telemedicine (*n* = 32), and a group with physicians who left telemedicine (*n* = 31). The 42-question survey was open between June and August of 2023. Participation was optional, anonymous, and voluntary.

**Results:**

In total, 93 participants completed the questionnaire. In terms of work–life balance, the best results were observed in the telemedicine group (4.63 vs. 4.35 in the control group). A high level of exhaustion was observed in 26.7% of the control group compared with 12.5% of the telemedicine group. Low professional fulfillment was present in 62.5% of participants in the telemedicine group vs. 46.7% of participants in the control group. Among the telemedicine physicians, 21.9% of participants were suspected of having depression.

**Discussion:**

Practicing telemedicine as a profession may have a positive impact on physicians’ work–life balance and burnout. Further research is necessary to explore the long-term effects of telemedicine on physicians’ mental health.

## Introduction

Remote health care is an excellent instrument for providing chronic care at home, reducing the oversaturation of hospitals and practices, and helping avoid the spread of infectious diseases. It may also be a key tool for making health care more sustainable and improving people’s health, leading to cost savings and greater efficiency.^1^ In addition, it facilitates health care delivery in conditions in which access to in-person visits is difficult, whether owing to areas geographically further away from health care centers, work or personal problems, or exceptional situations such as the COVID-19 pandemic.

Studies regarding patient interactions and satisfaction with telemedicine are, meanwhile, frequent. For instance, patients with chronic diseases, such as diabetes mellitus, obesity, or heart failure, report better quality of life when using wearables or digital health applications.^2–4^ In addition, acute health problems, such as back pain, urinary tract infections, and gastrointestinal infections, are treatable with telecare.^5–7^

While the benefits for patients are well documented, only a few studies have investigated how telemedicine affects physicians’ well-being and mental health. It is well known that the prevalence of burnout, depression, and other mental health issues among health care providers is significantly higher than that in the general population.^8^ Existing evidence shows the influence of poor mental health on patient care, medical mistakes, or career discontinuation, without forgetting the economic impact on health systems.^9^ There is a need to understand whether telemedicine benefits physicians’ work–life balance and mental health.

Most physicians had their first contact with telemedicine services during the pandemic. They generally described positive attitudes regarding the adoption of telemedicine and perceived more advantages in terms of flexibility and work–life balance.^10,11^ More flexibility at work and a better balance between professional and personal lives can help reduce burnout by boosting intrinsic motivation.^12^ However, as telemedicine is becoming the new standard of care, there is still a need to better understand its impact on physicians’ well-being and mental health. This study aimed to explore physician work–life balance, professional fulfillment, emotional exhaustion, and depression in telemedicine.

### Swiss Center for telemedicine—Medgate

Medgate, a Swiss Center for telemedicine, is a digital health company with 23 years of experience in telemedicine, covering all kinds of general medicine consultations, from acute health questions to prevention and chronic care management. Patients are treated 24 h a day via telephone, video, chat, and app by medical doctors trained in telemedicine. In 2022, Medgate provided an average of 1,778 consultations every day. During the study, 117 physicians practiced telemedicine at the center.

## Materials and Methods

Our study had a descriptive, comparative approach by using a cross-sectional questionnaire, which was open from June to August 2023. The questionnaire was distributed electronically among physicians working in Switzerland. The study had three groups: physicians who did not practice telemedicine (control group), physicians who practiced telemedicine in a large telemedicine center (experimental group), and physicians who left telemedicine. Physicians who left the telemedicine center and expressed their wish to stay in contact were e-mailed and encouraged to participate in the study as physicians who left telemedicine. Only those who left telemedicine altogether were eligible to take part in the study.

Participation was anonymous, optional, and voluntary, and consent was explicitly assumed by completing the questionnaire. We used the SoSci survey, a web-based software, to create the questionnaire (SoSci Survey GmbH, Munich). Our study did not require ethical approval from the Ethics Committee of Northwest and Central Switzerland (reference number 2023–00378).

The questionnaire included general demographic data (age, sex, experience in the current position) as well as validated questions on work–life balance, professional fulfillment, emotional exhaustion, and depression.

We assessed work–life balance using the German TriererKurzskala (TKS-WLB). It contains five statements rated on a Likert-scale form, from 1 (“do not agree”) to 6 (“completely agree.”) Examples of the items are: “I can meet the demands of my private life and the demands of my professional life equally well “or “I manage to achieve a good balance between stressful and restful activities in my life.” It is a validated scale on which higher punctuation indicates a better work–life balance.^13^

Professional fulfillment was assessed using the clinical version of the Stanford Professional Fulfilment Index. The index measures burnout and professional fulfillment. For our study, we only used the six items that measure separately professional fulfilment. Items like “I feel happy at work,” “My work is satisfying to me,” or “I feel in control when dealing with difficult problems at work” are scored from 0 to 4 (0 meaning “not at all true” and 4 “completely true.”) Physicians with average scores of ≥3 were more likely to be professionally satisfied.^14^

Emotional exhaustion was used as an approach for overall burnout since it is the primary feature of burnout and has a bigger predictive power than depersonalization.^15^ The nine-item Emotional Exhaustion scale from Maslach Burnout Inventory measures feelings of work exhaustion. Items from the Emotional Exhaustion scale are “I feel worn out at the end of a working day” or “I feel burned out because of my work.” The items are measured on a Likert-scale form from 0 points (“never”) to 6 points (“every day.”) A higher score correlates to greater burnout.^16^

The WHO-5 well-being test is among the most commonly used psychometric tools for assessing current general well-being and mental health. Its clinical validity for screening depression has been proven by numerous studies.^17–19^ It is an easy and economical way to screen for depression in primary health care. The 5-item scale measures the feelings of the past two weeks on a 5-point Likert scale. Items such as “I have felt calm and relaxed” or “My daily life has been filled with things that interest me” are rated from “all the time” to “at no time.” A value <13 points indicates poor well-being and can be used as an indication for a depression diagnosis.

### Statistical analysis

Standard descriptive statistics were used to characterize the questionnaire participants. To evaluate possible associations between variables, chi-square tests and variance analysis were used. Statistical significance was considered when *p*-value was <0.05. Owing to the size of the groups, a *post hoc* analysis was not performed. Statistical analyses were conducted using SPSS (version 29).

## Results

In total, 93 physicians participated in this study. (See Table 1).

**Table 1.** General Characteristics of the Study Population and Their Distribution in Subgroups

	Total	General practitioners	Telemedicine	Left telemedicine	
	*n* = 93	*n* = 30	*n* = 32	*n* = 31	*p* value
Age (years)	49.33 SD 9.2	50.13 SD 8.3	50.53 SD 10.7	47.32 SD 8.4	0.33
Gender					0.89
Female	62 (66.7%)	19 (63.3%)	22 (68.8%)	21 (67.7%)	
Male	31 (33.3%)	11 (36.7%)	10 (31.2%)	10 (32.3%)	
Partnership status					0.03
Yes	85 (91.4%)	29 (96.7%)	31 (96.9%)	25 (80.6%)	
No	8 (8.6%)	1 (3.3%)	1 (3.1%)	6 (19.4%)	
Children in household					0.06
Yes	68 (73.1%)	25 (83.3%)	25 (78.1%)	18 (58.1%)	
No	25 (26.9%)	5 (16.7%)	7 (21.9%)	13 (41.9%)	
Workload (in %)	72.0	70.9	59.5	75.3	0.009
Wished workload (in %)	64.3	63.3	52.7	72.1	0.00467
Years experience in the current position (mean average)	8.9	14.2	4.8	8.1	0.000035
How many night shifts do you have on average per month?					0.06
0–3	87 (93.5%)	29 (96.7%)	31 (96.9%)	27 (87.1%)	
3–7	5 (5.4%)	1 (3.3%)	1 (3.1%)	3 (9.7%)	
>7	1 (1.1%)	0	0	1 (3.2%)	
How many days per month do you work on weekends/holidays?					0.09
0–3	78 (84%)	28 (93.3%)	23 (71.9%)	27 (87.2%)	
3–7	13 (13.9%)	2 (6.7%)	9 (28.1%)	2 (6.4%)	
>7	2 (2.1%)	0	0	2 (6.4%)	
How satisfied are you with the current performance of your job?	5.6 SD 1.0	5.7 SD 0.9	5.6 SD 0.9	5.5 SD 1.2	0.88
(1—not at all; 7—completely satisfied)					
How would you describe your work–life balance?	5.3 SD 1.2	5.0 SD 1.0	5.7 SD 1.3	5.3 SD 1.3	0.39
(1—very bad; 7—very good)					
Do you have flexible working hours?	4.6 SD 1.7	4.3 SD 1.7	5.3 SD 0.7	4.2 SD 1.7	0.0014
(1—not at all true; 7—completely true)					

We contacted 321 general practitioners who did not practice telemedicine at the time of the study. From them, 30 decided to complete the questionnaire (9.34% response rate). Among 117 physicians practicing telemedicine at the Swiss center, 32 physicians participated (27.3% overall response rate) in this study. The group who left telemedicine comprised 31 participants from a total of 95 who were contacted (32.63% response rate).

**FIG. 1.**
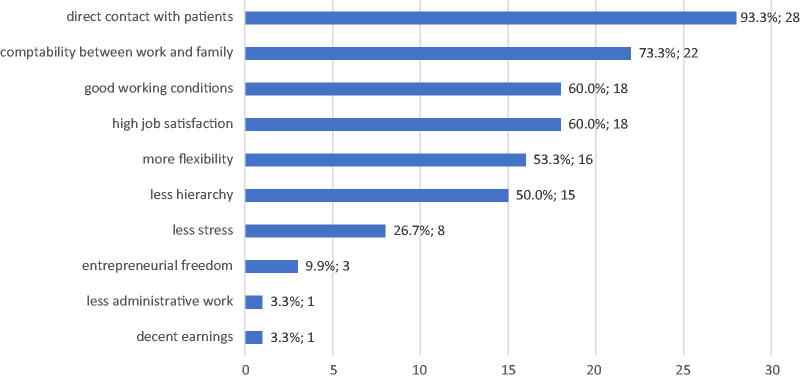
Reasons to work in a practice according to general practitioners, *n* = 30.

Physicians who did not practice telemedicine had a mean age of 50.13 years, were mostly female (63.3%), and were in a partnership (96.7%). The majority had children (83.3%), and they had a workload of 70.9% (around 33.6 h per week). Fourteen physicians responded that they wanted to reduce their workload (46.6%). When asked why they worked in a general practitioner (GP) practice, most of them mentioned direct contact with patients (93.3%), compatibility between family and work (73.3%), and good working conditions (60%) (see Fig. 1).

**FIG. 2.**
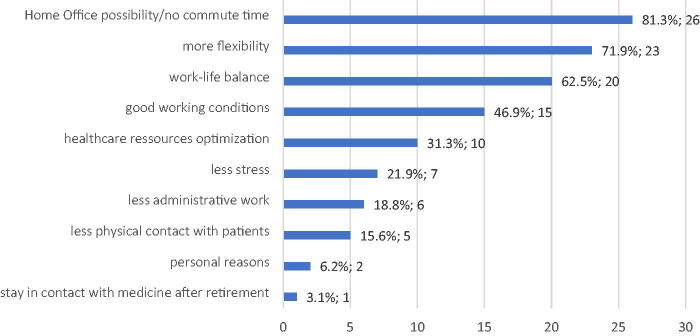
Reasons to practice telemedicine according to the experimental group, *n* = 32.

The telemedicine group had a mean age of 50.53 years; most physicians were female (68.8%) and were in a partnership (96.9%). In total 78.1% of participants had children, and they had a workload of 59.9% (approximately 28.8 h per week). Eleven physicians wanted to slightly reduce their workload (34.3%). The most frequent answers to the question of why they work in telemedicine were flexibility (71.9%), no commute time (81.3%), and work–life balance (62.5%). Curiously, 15.6% of physicians answered lack of direct contact with the patient (Fig. 2).

**FIG. 3.**
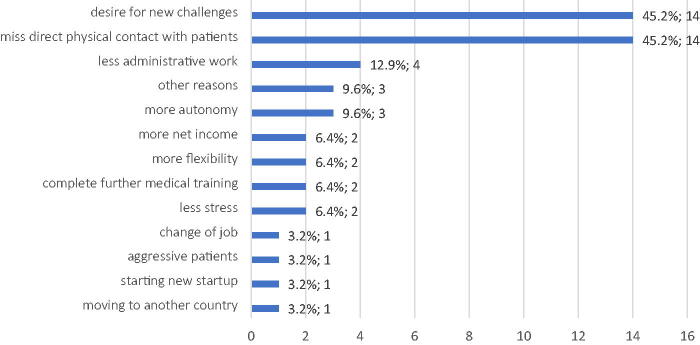
Reasons that led to stop practicing telemedicine according to the physicians who left the telemedicine center, *n* = 31.

The group of physicians who left telemedicine had a mean age of 47.32 years, and similar to other groups, females were more predominant in this group (67.7%). Almost all physicians were in a partnership (80.6%), and 58.1% had children. They had a workload of 75.3% (around 31.5 h a week), and nine physicians (29%) expressed their wish to reduce their working hours. When asked why they left telemedicine, two of the most frequent answers were missing direct contact with patients (45.2%) and a desire for new challenges (45.2%). (Fig. 3).

Partnership status, workload, wished workload, number of years’ experience in the position, and flexible hours differed statistically between the three groups (*p* 0.03, *p* 0.00983, *p* 0.00467, *p* 0.000035, and *p* 0.0014, respectively; Table 1).

### Work–life balance

**FIG. 4.**
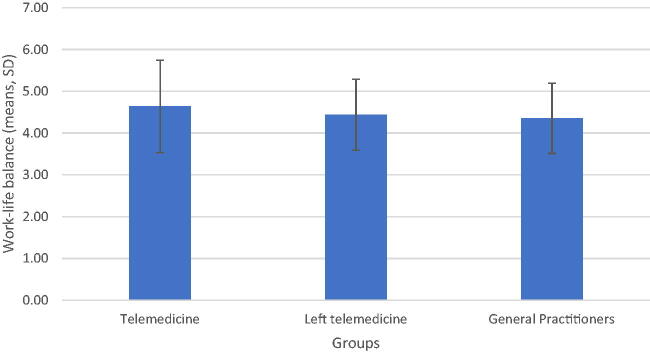
A comparison of mean values of work–life balance among the three groups.

In our study, the highest work–life balance score was found in the telemedicine group [4.64; standard deviation (SD) 1.10]. The GP group had the lowest score (4.35; SD 0.84), and the physicians who left telemedicine had the second largest mean (4.44; SD 0.84) (see Fig. 4).

To test the variability between group means, we used the *F*-test. Although the telemedicine group had the highest score in our study, the mean value of all groups (no longer telemedicine, telemedicine, and GPs) on the work–life balance scale did not differ significantly from one another [*F* (2.90) =1.663, *p* = 0.195].

### Professional fulfilment

Table 2 shows that 48.4% of the “left telemedicine” group, 62.5% of the “telemedicine” group, and 46.7% of the control group had low professional fulfillment. The percentage of professional fulfillment among groups did not differ statistically (chi-square value, 1.902; *p* 0.386).

**Table 2.** Professional Fulfilment among Groups

	Left telemedicine	Telemedicine	General practitioners	Total
Professional fulfilment				
Low (<3)				
Number	15	20	14	49
% within the group	48.4%	62.5%	46.7%	52.7%
High (>3)				
Number	16	12	16	44
% within the group	51.6%	37.5%	53.3%	47.3%
Total				
Number	31	32	30	93
% within the group	100.0%	100.0%	100.0%	100.0%

### Emotional exhaustion

In terms of emotional exhaustion, 26.7% of the GP group suffered from a high level of exhaustion, compared to 9.7% of the “left telemedicine” group and 12.5% of the telemedicine group.

**FIG. 5.**
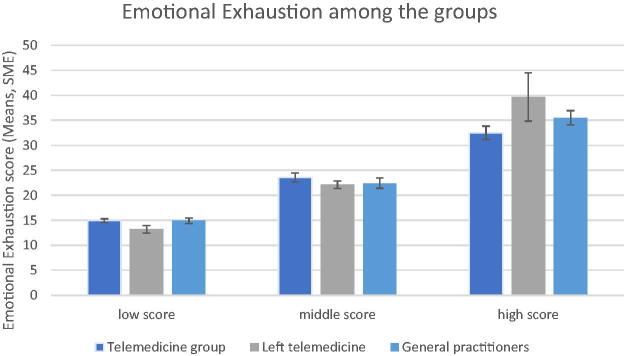
A comparison of emotional exhaustion scores among the three groups.

Additionally, it can be observed that approximately 67.8% of “left telemedicine,” 59.4% of “telemedicine” professionals, and 66.7% of general practitioners suffered from a medium or high level of professional exhaustion (Fig. 5 and Table 3).

**Table 3.** Emotional Exhaustion Frequencies in All Three Groups

	Left telemedicine	Telemedicine	General practitioners	Total
Emotional exhaustion				
Low (score, ≤17)				
Number	10	13	10	33
% within the group	32.2%	40.6%	33.3%	35.5%
Middle (score, 18–29)				
Number	18	15	12	45
% within the group	58.1%	46.9%	40%	48.4%
High (score, ≥30)				
Number	3	4	8	15
% within the group	9.7%	12.5%	26.7%	16.1%
Total				
Number	31	32	30	93
% within the group	100.0%	100.0%	100.0%	100.0%

We used the chi-square test to compare the means of emotional exhaustion. The differences between the groups were not statistically significant (chi-square value, 4.546; *p* 0.337).

### Depression

In total, 12.9% of the “left telemedicine” group, 21.9% of the “telemedicine” group, and 16.7% of the GP group were suspected of having depression (score < 13); these differences were not significant (chi-square value, 0.899; *p* 0.638; Table 4).

**Table 4.** Depression Scores among the Three Groups

	Left telemedicine	Telemedicine	General practitioners	Total
Suspicion of depression				
No suspicion (Score ≥13)				
Number	27	25	25	77
% within the group	87.1%	78.1%	83.3%	82.8%
Depression suspicion (<13 points)				
Number	4	7	5	16
% within the group	12.9%	21.9%	16.7%	17.2%
Total				
Number	31	32	30	93
% within the group	100.0%	100.0%	100.0%	100.0%

## Discussion

The present study aimed to assess physician work–life balance, professional satisfaction, emotional exhaustion, and depression in telemedicine. We found that although there were no statistically significant differences between the three groups, physicians practicing telemedicine had a higher work–life balance score and they were less emotionally exhausted than general practitioners who did not practice telemedicine. In terms of professional fulfillment and depression, in our study, physicians trained in telemedicine scored lower than their colleagues.

This study is one of the first, to the best of our knowledge, to explore well-being and mental health of physicians practicing telemedicine. Although the groups were relatively small, we had a normal distribution in every group. For instance, the telemedicine group was representative of the telemedicine center doctors in terms of age, sex, and workload, and we had a good overall response rate (27.35% in telemedicine physicians, 32.63% in left telemedicine physicians, and 9.34% in general practitioners).

Our study had, however, several limitations. First, we used a convenience sample with physicians who practiced or left telemedicine in a single center in Switzerland. This may limit the generalizability of the results. Second, we did not have a group of physicians who worked in a hybrid setting (telemedicine and practice). Third, our study had a cross-sectional design, and longitudinal approaches are needed to study the causality between telemedicine and mental health disorders.

Our results complement the results of other studies that have already suggested that telemedicine promotes a better work–life balance for physicians. DePuccio et al. found several benefits for physicians delivering health care remotely. In the semi-structured interviews conducted with general practitioners from a single academic medical center, physician work–life balance increased after using telemedicine. Flexibility and no commute time were also mentioned, like in our study.^20^ Moreover, a nationally representative questionnaire in Australia with 7,043 participants—including physicians who worked in primary care—found that telehealth improved job satisfaction and work–life balance compared to physicians who did not use it.^21^ Similar to our results, the study from Mayo Clinic about physicians’ experiences in telemedicine during the COVID-19 pandemic found that primary care physicians experienced less burnout thanks to telemedicine, and their work–life improved by around 36%.^22^

Perhaps because of its novelty, there were no studies about professional fulfillment in telemedicine. Most of the published studies focused on the general satisfaction with telemedicine and not on the professional realization. In terms of professional fulfillment, in our study, physicians practicing telemedicine showed lower levels of accomplishment. An explanation could be the limitations physicians face during such consultations: the impossibility of performing physical examinations and other complementary tests for a more precise diagnosis. As a result, these physicians refer a part of their patients, which may contribute to feeling less valued as professionals. Point-of-care tests and wearables in daily practice could be helpful for telemedicine doctors by supporting their clinical decisions and therefore improving professional satisfaction.

In our study, there were no statistical differences among the three groups regarding depression. A mixed-methods systematic review, however, found associations between physician burnout and depression, anxiety, and suicidality.^23^ In total, 45 studies were analyzed where the relationship between burnout and depression was measured, and in all these studies, the association appeared to be statistically significant.

An interesting point to mention from our study is that in every group, more than two-thirds of participants were female doctors. Because female physicians are still the main caregivers at home, and according to a study, this even worsened during the COVID-19 pandemic, telemedicine would allow them to reconcile family responsibilities with professional life.^24^

## Conclusion

This study suggests telemedicine may have a positive impact on physicians’ work–life balance and work exhaustion. Further studies are needed to confirm these findings and explore the long-term benefits of telemedicine on physicians’ mental health.

## Abbreviations Used

TKS-WLB: Trierer Kurz Scala-Work Life Balance
WHO: World Health Organization
SPSS: Statistical Package for Social Sciences
GP: General Practitioner
SD: standard deviation
